# Itraconazole associated quadriparesis and edema: a case report

**DOI:** 10.1186/1752-1947-5-140

**Published:** 2011-04-09

**Authors:** Rangaprasad L Karadi, David Gow, Mark Kellett, David W Denning, Ronan B O'Driscoll

**Affiliations:** 1 Medical Admissions Unit, Pontefract General Infirmary, Pontefract, WF8 1PL, UK; 2 Salford Royal NHS Foundation Trust, Hope Hospital, Stott Lane, Salford, M6 8HD, UK; 3 National Aspergillosis Centre, Education and Research Centre, University Hospital of South Manchester (Wythenshawe Hospital), Southmoor Road, Manchester M23 9LT, UK

## Abstract

**Introduction:**

Itraconazole is an anti-fungal agent widely used to treat various forms of mycosis. It is particularly useful in allergic bronchopulmonary aspergillosis and severe asthma with fungal sensitization. Side effects are uncommon and usually mild. Mild neuropathy is noted to occur very rarely. We present an unusual and, to the best of our knowledge, as yet unreported case of severe neuropathy and peripheral edema due to itraconazole in the absence of a concomitant risk factor.

**Case presentation:**

A 72-year-old Caucasian man was started on itraconazole following diagnosis of severe asthma with fungal sensitization. One month later he presented with severe bilateral ankle edema with an elevated serum itraconazole level. The itraconazole dose was reduced but his ankle edema persisted and he developed weakness of all four limbs. Itraconazole was completely stopped leading to improvement in his leg edema but he became bed bound due to weakness. He gradually improved with supportive care and neurorehabilitation. On review at six months, our patient was able to mobilize with the aid of two elbow crutches and power had returned to 5/5 in distal extremities and 4+/5 in proximal extremities. The diagnosis was established based on the classical presentation of drug-induced neuropathy and negative investigatory findings for any alternative diagnoses.

**Conclusion:**

We report the case of a patient presenting with an unusual complication of severe neuropathy and peripheral edema due to itraconazole. Clinicians should be alert to this association when encountered with neuropathy and/or edema in an itraconazole therapy recipient.

## Introduction

Itraconazole is an anti-fungal agent that was registered for use in 1991. It is widely used to treat several forms of mycosis. It was the first oral agent with activity against *Aspergillus *species [1]. It is also effective in allergic bronchopulmonary aspergillosis (ABPA) and in the newly described syndrome, severe asthma with fungal sensitization (SAFS) [2,3]. Side effects are uncommon (less than 7%) and usually mild [4-6]. Mild peripheral neuropathy is reported with a frequency of less than one in 10,000 [4]. Numerous case reports of itraconazole-enhanced vincristine neuropathy exist and are attributed to reduced vincristine metabolism by itraconazole [7-9]. We report a unique case of severe neuropathy due to itraconazole in the absence of a concomitant risk factor.

### Case presentation

**Figure 1:**
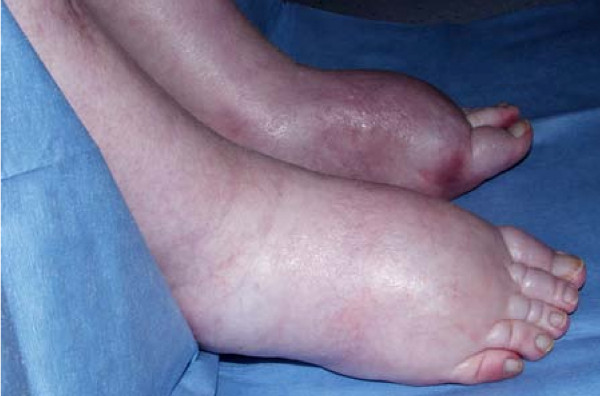
**Gross peripheral edema during itraconazole therapy**.

A 72 year-old Caucasian man had been on treatment for many years for severe asthma with relatively good exercise tolerance. Over a period of two years he developed increasing shortness of breath and productive cough necessitating assessment in a specialist immunology clinic. His total immunoglobulin E (IgE) was 680kIU/L and specific IgE against *Aspergillus fumigatus *was 14.6kUa/L. Precipitins against *A. fumigatus *were weakly positive (titre 1/2) without a peripheral eosinophilia. Computerized tomography revealed marked emphysema and mild bronchiectasis. Based on these results he was diagnosed with SAFS and itraconazole was commenced (Sporanox™ 200 mg twice daily). His only other medicines were formoterol fumarate (6 mg inhaler, one puff twice daily), fluticasone (500 mg inhaler, one puff twice daily) and nebulized salbutamol (2.5 mg twice daily). One month later he developed progressive bilateral ankle edema (Figure 1). The itraconazole level by bioassay was 17.5 mg/L (therapeutic range 5-15 mg/L) and the dosage was reduced to 200 mg once daily. A month later, peripheral edema persisted and he developed weakness of all four limbs. There were no signs of heart failure and the itraconazole level was 9.8 mg/L. Itraconazole was completely stopped leading to improvement in leg edema but limb weakness persisted. After six weeks he had become bed bound and he was referred to the tertiary neurology centre.

On admission, he had mild bilateral facial weakness. Tone was normal in his upper limbs but reduced in both his lower limbs. There was a grade 2-3/5 global weakness in both his lower limbs and grade 3/4 global weakness in both his upper limbs. He was areflexic in all four limbs. His vibration sense was absent up to his tibial tuberosity, joint position sense impaired up to the level of his metatarsal joint and pinprick sensation reduced to his mid-shin level.

Nerve conduction studies revealed globally attenuated motor and sensory responses without slowing, in keeping with a marked sensory and motor axonal polyneuropathy. Blood tests were mostly normal including a normal creatine kinase level. His lactase dehydrogenase level was mildly elevated at 490IU/L (reference range 105-333IU/L) and C-reactive protein level was 21 mg/L (reference range 0-10 mg/L). His thyroid function was normal and serum albumin level was modestly reduced, ranging from 23 to 29g/L during the illness. A sural nerve biopsy revealed mild axonal neuropathy and some evidence of regeneration, without evidence of vasculitis. A muscle biopsy (tibialis anterior) showed features consistent with an underlying neurogenic process (denervation related changes) and there was no evidence of vasculitis. Results from the examination of his cerebrospinal fluid (CSF) were normal with <1 leucocyte/μL, glucose 4.8 mmol/L and protein 0.4 g/L.

No immunoglobulin G (IgG) anti-nuclear, extractable nuclear, smooth muscle, mitochondrial, reticular, gastric parietal, para-neoplastic neuronal specific, acetylcholine receptor, perinuclear-staining anti-neutrophil cytoplasmic antibodies (p-ANCA) or cytoplasmic-staining anti-neutrophil cytoplasmic antibodies (c-ANCA) were detected in his blood. The erythrocyte sedimentation rate was 70 mm/h (range 0-65 mm/h). His repeat total IgE was 236kIU/L with *Aspergillus *specific IgE reduced to 6.84kUa/L (previously 14.6 kUa/L), consistent with a good response to itraconazole. Although in the initial differential, axonal Guillain-Barré syndrome (GBS) was considered, the results of the investigations, particularly CSF protein, and the subsequent disease course made the diagnosis of itraconazole-induced neuropathy most likely.

Our patient received supportive care initially followed by active neurorehabilitation. Gradual improvement occurred over a two-month period and our patient was discharged. He was reviewed in clinic six months after his discharge, and was able to mobilize with the aid of two elbow crutches. The power had returned to 5/5 in his distal upper and lower extremities and 4+/5 in his proximal upper and lower extremities.

## Discussion

Itraconazole is a synthetic triazole anti-fungal agent, which was first discovered in 1980 and licensed in 1991 for the treatment of invasive aspergillosis and several endemic mycoses, such as histoplasmosis and sporotrichosis. It has been extensively used for a variety of allergic, cutaneous and systemic mycoses and in treatment as well as prophylaxis of fungal infection in hematological and transplant patients [1].

In recent years, itraconazole has been increasingly used for treating ABPA and, very recently, the newly described syndrome SAFS [2,3]. Generally, itraconazole is well tolerated with only mild side effects reported [4-6]. In clinical studies the overall incidence of adverse events is in the order of 7% with short-term use, and up to 15% with long-term use - the gastrointestinal side effects being the most common [4,6]. Ankle edema is an uncommon complication of therapy with itraconazole [7]. It is reported in up to 4% of patients in clinical trials, being more common in patients receiving calcium channel blockers. The mechanism for this side effect remains unknown. Marked edema requiring drug suspension as seen in our case is a rare phenomenon and has been reported only once before [7]. Although, hypoalbuminemia (range 23-29g/L) was present, possibly aggravating the edema, this would not on its own be expected to give rise to the very marked edema that was observed in our case.

Itraconazole is a rare cause of peripheral neuropathy with a reported frequency of less than 1 out of 10,000 in the product data sheet [4]. Interestingly, there are several reports of itraconazole-enhanced vincristine neurotoxicity [8-10] and one report of painful neuropathy in a Type 1 diabetes mellitus patient [11]. However, severe neuropathy solely due to itraconazole without any concomitant risk factor, as in our case, has not been reported thus far.

Among the other azole drugs, there are at least four case reports of voriconazole causing painful peripheral neuropathy in the literature [12-14]. Posaconazole, despite being a highly lipophilic drug, has not been reported as an exclusive cause of neuropathy but evidence exists that it enhances vincristine-induced neurotoxicity [15,16].

Several features support the diagnosis in our case. Firstly, the temporal association between onset of ankle edema and neuropathy after commencing itraconazole, and gradual recovery upon stopping the drug, is very suggestive. The time and dose dependent pattern of axonal sensorimotor polyneuropathy, as seen in our case, is characteristic of drug-induced neuropathy [17]. Although the clinical features could represent the axonal variant of GBS, the normal CSF protein would be against this, as would the contemporaneous onset of ankle edema, a known side effect of itraconazole and not a feature of GBS. The only similarities noticed between vincristine associated neuropathy cases and our case are the presence of large fiber sensory loss, significant motor involvement, normal CSF findings and gradual recovery upon stopping the drug. The previous reports of neuropathy caused by combined treatment with vincristine and itraconazole mostly describe a form of rapidly progressing paresthesia and/or muscle weakness and paralytic ileus [8-10]. In all the above cases there was gradual neurological improvement upon stopping the causative drugs that is also evident in our case.

The possible mechanism for neuropathy due to itraconazole is speculative. Itraconazole is a cytochrome inhibitor with strong lipophilicity and high plasma protein binding ability (99%) [18,19]. The characteristic tissue penetrability manifests in a tissue concentration, two- to ten-fold higher than the plasma concentration. This has been deduced as the reason for its efficacy despite a very low plasma itraconazole concentration in some individuals. Conversely, in our patient, a high initial plasma itraconazole concentration would have led to substantial tissue saturation leading to neurotoxicity, and the subsequent slow clearance would account for the gradual recovery despite early drug discontinuation [20]. An additional mechanism relevant to our case is the 'active efflux system' in brain described by Miyama and colleagues [21]. Accordingly, itraconazole if present at low concentration in brain undergoes faster elimination and when present at high concentration undergoes slower elimination. The latter scenario would be pertinent to our case.

It is notable in our case that initial itraconazole levels were high despite standard dosing. This is explained by the fact that itraconazole levels are not only dose dependent but can significantly vary in individuals receiving the same dose, probably reflecting its hepatic metabolism and enterohepatic circulation [22]. Hence, initial dosing cannot predict the steady state plasma concentration. It is conceivable that higher levels would lead to higher tissue saturation and are more likely to cause neuropathy. Consequently, we would advise monitoring the levels following initial dosing with this drug and further monitoring if any dose increments are made.

## Conclusion

We report, what we believe to be the first case of severe neuropathy and ankle edema due to itraconazole in the absence of a concomitant risk factor. Clinicians should be alert to this association when encountering neuropathy and/or edema in an itraconazole therapy recipient.

## Abbreviations

ABPA: allergic bronchopulmonary aspergillosis; c-ANCA: cytoplasmic staining antineutrophil cytoplasmic antibodies; CSF: cerebrospinal fluid, IgE: immunoglobulin E; IgG: immunoglobulin G; p-ANCA: perinuclear staining antineutrophil cytoplasmic antibodies; SAFS: severe asthma with fungal sensitization.

## Consent

Written informed consent was obtained from the patient for publication of this case report and any accompanying images. A copy of the written consent is available for review by the Editor-in-Chief of this journal.

## Competing interests

DWD holds founder shares in F2G Ltd. and Myconostica Ltd., both University of Manchester spin-out companies, and has received grant support from F2G as well as the Fungal Research Trust, the Wellcome Trust, the Moulton Trust, The Medical Research Council, The Chronic Granulomatous Disease Research Trust, the National Institute of Allergy and Infectious Diseases, National Institute of Health Research and the European Union, AstraZeneca and Basilea. He continues to act as an advisor and consultant to F2G and Myconostica, as well as for other companies over the last five years including Basilea, Vicuron (now Pfizer), Pfizer, Schering Plough, Nektar, Daiichi, Astellas, Gilead and York Pharma. He has been paid for talks on behalf of Schering, Astellas, Merck, Dainippon and Pfizer. MK has received sponsorship from Orion Pharma, GlaxoSmithKline, UCB Pharma and Boehringher Ingelheim to attend conferences or meetings. RLK, DG and BRO declare that they have no competing interests.

## Authors' contributions

RLK reviewed the patient's clinical data, performed the literature search and wrote the initial draft. DG, MK, DWD and BRO reviewed the initial draft and finalized the manuscript. All authors read and approved the final manuscript.
